# The great outdoors? Exploring the mental health benefits of natural environments

**DOI:** 10.3389/fpsyg.2014.01178

**Published:** 2014-10-21

**Authors:** David G. Pearson, Tony Craig

**Affiliations:** ^1^School of Psychology, University of AberdeenAberdeen, UK; ^2^Social, Economic and Geographical Sciences, The James Hutton InstituteAberdeen, UK

**Keywords:** attention restoration theory, perceived restorative properties, restorative environments, natural environments, cognitive fatigue, health benefits

There is growing evidence to suggest that exposure to natural environments can be associated with mental health benefits. Proximity to greenspace has been associated with lower levels of stress (Thompson et al., 2012) and reduced symptomology for depression and anxiety (Beyer et al., 2014), while interacting with nature can improve cognition for children with attention deficits (Taylor and Kuo, 2009) and individuals with depression (Berman et al., 2012). A recent epidemiological study has shown that people who move to greener urban areas benefit from sustained improvements in their mental health (Alcock et al., 2014). In this paper we critically review evidence indicating that such mental health benefits are associated with the so-called “restorative” properties of natural environments. In particular we focus on the claim that interaction with (or just passive *perception* of) natural scene content can be linked to the restoration of limited-capacity attentional resources, in comparison to similar exposure to urban or built scene content.

## What makes an environment restorative?

Attentional restoration theory (ART) is an influential framework first proposed by Kaplan and Kaplan (1989) that claims urban environments suffer from an excess of bottom-up stimulation that serves to dramatically capture attention. People exposed to urban environments are forced to use their attention to overcome the effects of constant stimulation (described as *hard fascination*), and this in turn over time induces cognitive fatigue. In contrast, natural environments benefit from what the Kaplan's term *soft fascination*, which refers to scene content that automatically captures attention while simultaneously eliciting feelings of pleasure. Although there is no direct equivalent of hard and soft fascination in the cognitive attentional literature, the terms have been related to the concept of voluntary and involuntary attention (James, 1892). The process of soft fascination is seen to reduce the demand on executive-based attention, thereby allowing greater restoration of depleted attentional resources in comparison to the perception of urban environments (Kaplan, 1995, 2001). Kaplan and Berman (2010) have proposed that natural environments can restore directed attention; a common resource that supports both executive functioning and self-regulation processes in cognition (Baumeister et al., 1998; Norman and Shallice, 2000).

Other important features of restorative environments identified by ART include the experience of *being* away, in which a person feels a sense of escape from the stressful demands of daily life, and *extent*, in which a perception of vastness, and connectedness in an environment helps promote related experiences of “being away.” Studies supporting ART have demonstrated improved performance on attention-demanding tasks following time spent in natural environments (e.g., Hartig et al., 1991; Berman et al., 2008). Intriguingly, attention restoration effects are also observed after participants simply watch films or photographs that depict natural scene content (van den Berg et al., 2003; Berto, 2005), implying that direct physical engagement with nature may be unnecessary to promote positive restoration effects.

ART has been widely cited in the literature as supporting superior health benefits of natural environments in comparison to urban environments. However, an important feature of ART that distinguishes it from more psycho-evolutionary frameworks such as that proposed by Ulrich (1983) is that the key informational elements such as *being away* and *fascination* that help determine restorative environments need not be *uniquely* associated with natural environments alone. For example, man-made structures such as monasteries can also be considered restorative environments (Ouellette et al., 2005). Although Korpela et al. (2001) have argued that restorative experiences are overrepresented in natural environments when students are asked to describe their favorite places, they notably allow within their definition of “natural” reference to man-made features such as “cottage surrounded by trees next to a lake” (p. 580). Residential and leisure environments (e.g., museums, art galleries) have also been claimed to reduce demands placed on executive attention and thereby promote psychological restoration (Staats, 2012). Findings such as these suggest that restorative properties associated with exposure to natural environments may not derive from intrinsic properties of the scene content itself, but instead from much broader contextual, and associative factors. The majority of studies reported in the literature tend to compare natural scenes against urban scenes, whether in the form of physical real-world environments or virtual depictions in films and photographs. Critically, we argue that careful stimuli selection for these types of studies is vitally important. There is a tendency to treat the categories of “natural” and “urban/built” as being more clearly defined and identifiable than may actually be the case in every-day judgments (van der Jagt et al., 2014). Indeed, in a study highlighting the importance of water in stimuli selection, White et al. (2010) point out that many studies in this area have demonstrated a bias toward the inclusion of aquatic scenes in the positive-natural category, and that urban scenes containing water were just as likely to elicit positive responses.

## The relationship between perceived restorative properties and attention-restoration

Studies examining the relationship between exposure to natural scenes and attention restoration have used a variety of different tasks and measures of cognitive function in order to garner experimental evidence of any “attention restoration effect.” Many studies involve inducing mental fatigue for a group of participants and then comparing that group with a non-fatigued group. For example, Hartig et al. (2003) induced mental fatigue by asking half of their participants to carry out a sequence of Stroop and binary classification tasks. van den Berg et al. (2003) played participants fragments of a distressing film to ensure that participants were suitably in need of restoration prior to carrying out the experiment, and other studies have used naturalistic fatigue induction protocols, such as sampling participants after lectures or exams (e.g., Hartig and Staats, 2006; Karmanov and Hamel, 2008), or have asked people to “imagine” that they are attentionally fatigued (e.g., Staats and Hartig, 2004).

Notwithstanding the method used to induce attention fatigue, it is important to distinguish between what might be termed “directed attention fatigue”—indicated by a lowering of performance on attention-demanding tasks, and other forms of fatigue such as those related to sleepiness, emotional stress or boredom. For example, the aforementioned study by van den Berg et al. (2003) induced stress via an emotionally distressing movie, but then went on to test for attention-restoration via a concentration index. A wide range of measures of cognitive function have been used to detect differences in attention-restoration, including: the backwards digit span task (e.g., Berman et al., 2008), the Necker cube pattern control task (e.g., Hartig et al., 2003), the symbol digit modalities test (e.g., Tennessen and Cimprich, 1995), and the attention network task (e.g., Berman et al., 2008). It is potentially problematic in drawing meaningful conclusions from studies if different concepts of what constitutes “stress” are conflated, or if alternative explanations such as motivation or mood effects are not sufficiently controlled for.

We also argue that an important distinction needs to be made between the perceived restorative properties of images or environments, and levels of *actual restoration* following direct experience with an environment. Various efforts have been made to develop scales that allow the perceived restorative potential of environments to be assessed, including the Perceived Restorativeness Scale (Hartig et al., 1997), the Restorative Components Scale (Laumann et al., 2001), and the Perceived Restorative Characteristics Questionnaire (Pals et al., 2009). All of these measures attempt to measure the restorative components of environments as specified by ART, and they are useful insofar as they help to understand the underlying dimensions of environmental preference and associated behavioral choice. However, we argue that the extent to which perceived restoration predicts *actual* restoration is undervalued in the literature. There is an assumption that individuals have sufficiently high metacognitive understanding of their own cognitive processes that they can accurately estimate how different environments will affect them. There is surprisingly little direct evidence for this, and in other areas of cognition such as memory or perception people can be poor at accurately predicting their own performance (e.g., Bona and Silvanto, 2014; Roediger and DeSoto, 2014). Furthermore, if ART is correct and restoration results from an interaction between directed attention and the intrinsic properties of an environment, such restoration should occur irrespective of whether it has been previously “perceived” or not. If perceived restoration measures are used in the absence of any hard evidence for related cognitive effects, we argue there is a potential risk that the true restorative potential of natural, and urban environments could be misrepresented.

## What is the optimal form of interaction with restorative environments?

Although Stamps (1990) suggests that static photographs are sufficiently valid representations in allowing aesthetic judgments to be made, forming an aesthetic judgment of an image is somewhat different to judging the wider qualities of the environment depicted in the image. That said, even when focussing on aesthetic judgments, simply adding a degree of dynamism can significantly affect the ratings of preference for scenes (Heft and Nasar, 2000). If simply adding dynamism to environmental depictions affects preference, then it seems reasonable to expect that greater degrees of interaction will play an important role in terms of the mechanisms that environments can be thought of as facilitating psychological restoration. Do people need to physically interact with nature to receive the apparent health benefits, or is passive visual exposure to films sufficient? de Kort et al. (2006) suggest that “immersion” is an important component of the person-environment interaction in terms of psychological restoration, and that virtual environments can potentially be used to create a reasonable analog in order to study these important questions more fully. However, the closer that technology allows studies to approximate visual and experiential reality, the more questions can be asked about what is missing from such simulations in comparison to real environments. When studies suggest that large television displays (Friedman et al., 2008) or large wall murals (Felsten, 2009) could potentially form part of a future built environment where windows are not possible (e.g., in basements), or where the view from a window is rather mundane, the implications for the preservation of urban greenspace could be quite stark if simulations are judged to have the same restorative properties as real environments. However, studies that have directly compared simulated and real environments on their restorative properties suggest they are not equivalent (Martens and Bauer, 2008; Kjellgren and Buhrkall, 2010). Notwithstanding some of the methodological challenges with carrying out these types of research studies, they serve an additionally important function insofar as they force us to think more about the deeper philosophical issues at the heart of human-nature relationships.

## Closing comments

The growing trend for urbanization means the majority of the world's population are spending less time exposed to natural environments. This trend has potentially very serious implications for health if exposure to natural environments is causal to short-term recovery from stress or mental fatigue, and to overall long-term improvements in health and well-being. If the postulated causal relationship between natural environments and mental health is correct, then increasing accessibility to well-maintained greenspace and instigating behavior change programs that encourage greater interaction with nature could deliver substantial short and long-term benefits to mental health. However, much of the existing evidence base is based on an arguably too simplistic “natural” and “built” dichotomy. The importance of people's attitudes and beliefs toward health and the environment, and how these may interact with behavioral and physiological responses, is in particular poorly represented by the existing evidence base.

In closing, we therefore suggest that there is a pressing need for more empirical research that has the specific aim of establishing: (1) which properties of environments make them more or less “restorative”; (2) the relationship between perceived restorative properties of an environment and objective measures of improved cognitive function; and (3) the optimal form of interaction with restorative environments that is most likely to lead to mental health and well-being benefits.

### Conflict of interest statement

The authors declare that the research was conducted in the absence of any commercial or financial relationships that could be construed as a potential conflict of interest.
